# A Novel Divergent Geminivirus Identified in Asymptomatic New World Cactaceae Plants

**DOI:** 10.3390/v12040398

**Published:** 2020-04-03

**Authors:** Rafaela S. Fontenele, Andrew M. Salywon, Lucas C. Majure, Ilaria N. Cobb, Amulya Bhaskara, Jesús A. Avalos-Calleros, Gerardo R. Argüello-Astorga, Kara Schmidlin, Anthony Khalifeh, Kendal Smith, Joshua Schreck, Michael C. Lund, Matias Köhler, Martin F. Wojciechowski, Wendy C. Hodgson, Raul Puente-Martinez, Koenraad Van Doorslaer, Safaa Kumari, Christian Vernière, Denis Filloux, Philippe Roumagnac, Pierre Lefeuvre, Simone G. Ribeiro, Simona Kraberger, Darren P. Martin, Arvind Varsani

**Affiliations:** 1 The Biodesign Center for Fundamental and Applied Microbiomics, Arizona State University, Tempe, AZ 85287, USA; rafasfontenele@asu.edu (R.S.F.); ilaria.cobb@gmail.com (I.N.C.); amulyabhaskara@gmail.com (A.B.); kara.schmidlin@asu.edu (K.S.); akhalif5@asu.edu (A.K.); krsmit39@asu.edu (K.S.); jrschrec@asu.edu (J.S.); mclund2@asu.edu (M.C.L.); simona.kraberger@asu.edu (S.K.); 2 School of Life Sciences, Arizona State University, Tempe, AZ 85287, USA; Martin.Wojciechowski@asu.edu; 3 Desert Botanical Garden, Phoenix, AZ 85008, USA; asalywon@dbg.org (A.M.S.); lmajure@floridamuseum.ufl.edu (L.C.M.); whodgson@dbg.org (W.C.H.); rpuente@dbg.org (R.P.-M.); 4 Florida Museum of Natural History, University of Florida, Gainesville, FL 32611, USA; 5 The University of British Columbia, Vancouver, BC V6T 1Z4, Canada; 6 Center for Research in Engineering, Science and Technology, Paradise Valley High School, 3950 E Bell Rd, Phoenix, AZ 85032, USA; 7 División de Biología Molecular, Instituto Potosino de Investigación Científica y Tecnológica, A.C., Camino a la Presa de San José 2055, Lomas 4ta Secc, San Luis Potosi 78216, S.L.P., Mexico; jesus.avalos@ipicyt.edu.mx (J.A.A.-C.); grarguel@ipicyt.edu.mx (G.R.A.-A.); 8 Departamento de BotânicaPrograma de Pós-Graduação em Botânica, Universidade Federal do Rio Grande do Sul, Porto Alegre, RS 91501970, Brazil; matias.k@ufrgs.br; 9 School of Animal and Comparative Biomedical Sciences, Department of Immunobiology, BIO5 Institute, and UA Cancer Center, University of Arizona, Tucson, AZ 85721, USA; vandoorslaer@email.arizona.edu; 10 International Center for Agricultural Research in the Dry Areas (ICARDA), Terbol Station, Beqa’a, Zahle, Lebanon; s.kumari@cgiar.org; 11 CIRAD, BGPI, 34398 Montpellier, France; christian.verniere@cirad.fr (C.V.); denis.filloux@cirad.fr (D.F.); philippe.roumagnac@cirad.fr (P.R.); 12 BGPI, INRAE, CIRAD, SupAgro, Univ Montpellier, 34398 Montpellier, France; 13 CIRAD, UMR PVBMT, F-97410 St. Pierre, La Réunion, France; pierre.lefeuvre@cirad.fr; 14 Embrapa Recursos Genéticos e Biotecnologia, Brasília, CEP 70770-917, Brazil; simone.ribeiro@embrapa.br; 15 Computational Biology Division, Department of Integrative Biomedical Sciences, Institute of Infectious Diseases and Molecular Medicine, University of Cape Town, Observatory, Cape Town 7925, South Africa; darrenpatrickmartin@gmail.com; 16 Center for Evolution and Medicine, Arizona State University, Tempe, AZ 85287, USA; 17 Structural Biology Research Unit, Department of Clinical Laboratory Sciences, University of Cape Town, Cape Town 7925, South Africa

**Keywords:** geminivirus, Cactoideae, Opuntioideae, ssDNA virus, cochineal insects

## Abstract

Cactaceae comprise a diverse and iconic group of flowering plants which are almost exclusively indigenous to the New World. The wide variety of growth forms found amongst the cacti have led to the trafficking of many species throughout the world as ornamentals. Despite the evolution and physiological properties of these plants having been extensively studied, little research has focused on cactus-associated viral communities. While only single-stranded RNA viruses had ever been reported in cacti, here we report the discovery of cactus-infecting single-stranded DNA viruses. These viruses all apparently belong to a single divergent species of the family *Geminiviridae* and have been tentatively named Opuntia virus 1 (OpV1). A total of 79 apparently complete OpV1 genomes were recovered from 31 different cactus plants (belonging to 20 different cactus species from both the Cactoideae and Opuntioideae clades) and from nine cactus-feeding cochineal insects (*Dactylopius* sp.) sampled in the USA and Mexico. These 79 OpV1 genomes all share > 78.4% nucleotide identity with one another and < 64.9% identity with previously characterized geminiviruses. Collectively, the OpV1 genomes display evidence of frequent recombination, with some genomes displaying up to five recombinant regions. In one case, recombinant regions span ~40% of the genome. We demonstrate that an infectious clone of an OpV1 genome can replicate in *Nicotiana benthamiana* and *Opuntia microdasys.* In addition to expanding the inventory of viruses that are known to infect cacti, the OpV1 group is so distantly related to other known geminiviruses that it likely represents a new geminivirus genus. It remains to be determined whether, like its cactus hosts, its geographical distribution spans the globe.

## 1. Introduction

With the exception of a single species, *Rhipsalis baccifera* (Sols.) Stearn, which is also found in some tropical areas of the Old World, cacti are endemic to the Americas [1]. Cacti have undergone adaptive radiations across a wide variety of edaphically dry environments [1,2], which, together with high degrees of phenotypic diversification within the family, have yielded a broad range of morphological forms [3,4]. Phylogenetic relationships in the family are relatively well-known, and four principle clades have been recovered in analyses (*Leuenbergeria, Pereskia*, Cactoideae + *Maihuenia*, and Opuntioideae) [2,5,6]. Cacti are culturally, economically and ecologically important [7]. Since Europeans first arrived in the Americas, cacti have been transported throughout the world [1]: to be grown primarily as ornamentals, but also as a crop for their fruit and stems (known as nopales) and the farming of cochineal insects (*Dactylopius* spp.), the latter of which are members of the order Hemiptera, used for the production of the carminic acid dye [8].

In 1885, the first evidence of spindle-like structures associated with a virus infection was described from cacti in the genus *Epiphyllum* [9]. Since then, a handful of viruses have been identified in other members of the Cactaceae, all of which belong to the single-stranded RNA virus families *Alphaflexiviridae*, *Betaflexiviridae*, *Puribunyaviridae*, *Tombusviridae* and *Virgaviridae* [10,11,12,13,14,15,16,17,18]. To our knowledge, no plant-infecting DNA viruses (i.e., viruses belonging to the families *Geminiviridae*, *Nanoviridae,* and *Caulimoviridae*) have ever been found to infect cacti.

High-throughput sequencing (HTS) technologies have led to a dramatic increase in the discovery of novel viruses across ecosystems, and have broadly expanded our knowledge of plant-infecting virus diversity [19,20]. The impacts of these technologies on plant virus discovery are evident within the family *Geminiviridae,* a family of plant viruses for which HTS-based virus discovery projects are uncovering a growing number of divergent lineages. In addition to the nine recognized geminivirus genera—*Becurtovirus*, *Begomovirus*, *Capulavirus*, *Curtovirus*, *Eragrovirus*, *Grablovirus*, *Mastrevirus*, *Topocuvirus* and *Turncurtovirus*; [21,22]—four of which were established based on viruses discovered in large-scale HTS-based virus discovery projects, it is likely that multiple new genera will need to be formed to accommodate 12 other, currently unassigned, divergent geminivirus lineages [23,24,25,26,27,28,29,30,31,32].

Although many of the known geminiviruses cause severe economic losses in a variety of crops (i.e., tomato, maize, cotton, cassava and bean plants) [33,34], many of the newly discovered geminiviruses seem to produce either no symptoms or only very mild symptoms, in the host species from which they were isolated [25,31,35,36,37].

Besides prompting the founding of new geminivirus genera, newly discovered divergent geminivirus lineages are illuminating the deep evolutionary history of this family. The circular single-stranded DNA genomes of the known geminiviruses are encapsidated in twinned icosahedral particles [38] and encode up to seven genes that are bi-directionally transcribed. The only two genes that are detectably conserved across all of these divergent lineages are a replication associated protein gene (*rep*) and a capsid protein gene (*cp*). In addition to these two genes, three others, a replication enhancer protein gene (*ren*), a C4 gene (which encodes a symptom determinant and/or a silencing suppressor), and a transactivation protein gene (*trap*), are possibly conserved across the genera *Begomovirus*, *Curtovirus*, *Eragrovirus*, *Topocuvirus* and *Turncurtovirus*, although in some cases these genes are only putative homologs [21,39,40,41]. Although movement protein genes (*mp*) appear to occur in all known geminivirus genomes [40,41], there is commonly no detectable homology between the movement proteins (MPs) of viruses in the different geminivirus genera.

Geminiviruses are transmitted by a range of insect vectors in the order Hemiptera. In most cases, only one or a few very closely related vector species in a single genus transmit these viruses in each of the different geminivirus genera. Becurtoviruses, curtoviruses, and turncurtoviruses are known to be transmitted by leafhoppers in the genus *Circulifer*, begomoviruses by whiteflies in the genus *Bemisia,* topocuviruses by treehoppers in the genus *Micrutalis*, grabloviruses by treehoppers in the genus *Spissistilus*, and capulaviruses by aphids in the genus *Aphis* [21,33,42,43,44]. In the case of mastreviruses, however, different virus species are transmitted by insects belonging to different leafhopper species in a number of insect genera including *Cicadulina, Orosius*, *Psammotettix,* and *Nesoclutha* [45].

Although geminivirus research in the past has primarily focused on viruses that are major pathogens of cultivated plants, much recent attention has been given to geminiviruses that circulate within natural ecosystems, especially those at agro-ecological interfaces [46,47,48,49]. The spill-over of viruses between agricultural and natural ecosystems can significantly impact both the preservation of natural ecosystems [50,51] and the emergence of new crop pathogens from these ecosystems [52,53,54].

Here, we describe the characterization of a divergent geminivirus lineage found to infect different cactus species and multiple genera (*Opuntia* spp., *Cylindropuntia* spp. and *Lophocereus schottii*) in the USA and Mexico. The viruses within this lineage have tentatively been grouped with a species named Opuntia virus 1 (OpV1). Infectivity assays involving *Nicotiana benthamiana* and three *Opuntia* spp. confirmed that OpV1 was able to asymptomatically infect *N. benthamiana* and *O. microdasys*.

## 2. Materials and Methods

### 2.1. Sample Collection and Processing

A total of 527 Cactaceae plant samples (Supplementary Data 1) from the Cactoideae and Opuntioideae clades were collected in Argentina (*n* = 14), Bolivia (*n* = 8), Brazil (*n* = 8), Cuba (*n* = 1), Curaçao (*n* = 1), Dominican Republic (*n* = 2), France (*n* = 20), Haiti (*n* = 2), Lebanon (*n* = 1), Morocco (*n* = 1), Mexico (*n* = 31), Paraguay (*n* = 3), Reunion (19), Spain (*n* = 6), Tunisia (*n* = 10), Uruguay (*n* = 5), the United States (*n* = 394) and Venezuela (*n* = 1). Of the cactus samples from the USA, 134 were collected from the cactus collection at the Desert Botanical Garden in Phoenix, Arizona (USA). In addition, 25 non-cactus samples (from the Alliaceae, Amaranthaceae, Apiaceae, Asteraceae, Cucurbitaceae, Lamiaceae, Laureaceae, Malvaceae, Oleaceae and Solanaceae flowering plant families) were also collected from the Desert Botanical Garden in Phoenix, Arizona. Samples were collected using a 3 mm biopsy punch (Robbins Instruments, Chatham, NJ, USA) or scalpel blades. Although none of the sampled cacti were observed to have obvious infection symptoms, 61 plants were infested with cochineal insects (*Dactylopius* sp.). Insects from these 61 plants were also collected (see Supplementary Data 1 for details of all the samples analyzed). All samples were stored at −20 °C or dried on silica until processing.

Total DNA was extracted from cactus tissue samples using either the GeneJET Plant Genomic DNA Purification Kit (Thermo Fisher Scientific, Waltham, MA, USA) or DNeasy Plant Mini Kit (Qiagen, Hilden, Germany) according to the manufacturer’s instructions. The cochineal insects (cohorts of 5–10 from a colony) were ground in 200 µl of SM Buffer (0.1 M NaCl, 50 mM Tris/HCl-pH 7.4, 10 mM MgSO_4_) and subsequently centrifuged for 5 min at 10,000 rpm to pellet cellular material. The supernatant was then used to isolate DNA using the High Pure Viral Nucleic Acid Kit (Roche Diagnostics, Indianapolis, IN, USA). Both plant total DNA and cochineal insect purified viral DNA from each sample were used in a rolling circle amplification (RCA) reaction with the TempliPhi™ kit (GE Healthcare, Chicago, IL, USA), as described by Shepherd et al. [55].

### 2.2. High Throughput Sequencing and Genome Assembly

Aliquots of the RCA product of each sample were pooled (8 to 10 samples per pool) based on sampling location, and sequenced on an Illumina HiSeq 4000 platform (paired-end 2 × 100 bp) at Macrogen Inc. (Seoul, Korea). Raw reads were *de novo* assembled using SPAdes v. 3.12.0 [56] and the resulting contigs were analyzed using BLASTx [57] against a GenBank viral RefSeq protein database [58]. For contigs with a detectable homology (E-value of < 10^−5^) to known geminiviruses, abutting primers were designed (OpV1_F 5′-GGG CCC CAA TAA GTT CTT TCC AAT GTT TTA GCT TT-3′ and OpV1_R 5′-AAA GAG ACT GGC AAA GCA ACT GTA AAT ACG GCA AG-3′) to recover potentially full-length virus genomes from plant and insect samples. The primers were used to amplify the geminivirus genomes using KAPA HiFi HotStart DNA polymerase (KAPA Biosystems, USA), following the manufacturer’s thermal cycling condition recommendations. Amplicons were resolved in 0.7% agarose gel and those with a size of between 2.5 and 3.5 kb (the expected size-range of geminivirus genomes or genome components) were excised, gel-purified and cloned in the pJET1.2 cloning vector (Thermo Fisher Scientific, Waltham, MA, USA). Cloned amplicons were Sanger sequenced by primer walking at Macrogen Inc. (Seoul, South Korea). Genome assemblies and annotations were performed using Geneious 11.1.5 [59].

### 2.3. Infectivity Assays

One Opuntia-derived geminivirus isolate, OpV1 DBG14_1 (GenBank accession # MN100000) recovered from *O. echios* var. *echios* sampled from the Desert Botanical Garden (Phoenix, AZ, USA) was chosen for the construction of an infectious OpV1 clone. OpV1 F/R primers were phosphorylated using T4 kinase (New England Biolabs, Ipswich, MA, USA) and subsequently used to amplify the genome from OpV1 DBG14_1. The amplified genome was self-ligated using T4 DNA ligase (Thermo Fisher Scientific, Waltham, MA, USA) to generate a circular genome, which was subsequently amplified by RCA with the TempliPhi™ kit (GE Healthcare, Chicago, IL, USA). The RCA product was then digested with either *Hind*III to generate a linearized full genome copy (FGC; 2945 nt in length), or with both *Hind*III and *Bam*HI to generate a near full-length genome copy (nFGC; 2750 nt in length). The FGC and nFGC were individually cloned into the *Hind*III and/or *Bam*HI restriction enzyme sites of the vector pBlueScript-KS, and Sanger sequenced by primer walking at Macrogen Inc. (Seoul, South Korea). The FGC and nFGC were then cloned in the *Hind*III/*Bam*HI digested pGTV-kan [60] binary vector and used to transform *Escherichia coli* XL1 Blue. To confirm two copies had ligated in tandem, clones were tested by digesting them with *Bam*HI. A clone containing tandemly cloned FGC and nFGC was then used to transform *Rhizobium radiobacter* (synonymous species name for *Agrobacterium tumefaciens*) GV3101. A glycerol stock of this was prepared and stored at −80 °C.

Infection assays were performed on *N. benthamiana*, *O. ficus-indica*, *O. microdasys*, *O. engelmannii*, and *O. santa-rita*. *Rhizobium*-mediated OpV1 infections of *N. benthamiana* were performed in three replicates, with 18 inoculated plants in two replicates and seven in the third, including two negative controls (non-inoculated plants) in each replicate. Five opuntia plants for each species were *Rhizobium*-inoculated, and one plant was used as a negative control. For the *Rhizobium*-inoculations, *R. radiobacter* was grown for 20 h in Luria broth with kanamycin (50 µg/mL) and rifampicin (50 µg/mL). The culture was then centrifuged for 10 min at 4600 rpm to pellet the cells before resuspension in MES buffer (10 mM MES hydrate and 10 mM MgSO_4_ hepta-hydrate) with acetosyringone 150 µM to an OD of 1.0.

The seven inoculated *Nicotiana benthamiana* plants from the third infection assay were used for Southern blot analysis. We also included two negative control plants (non-inoculated). Total DNA was extracted from the *N. benthamiana plants* as described in Section 2.1 and 5 µg total DNA from each plant and a positive control (5 ng of OpV1 PCR amplicon of the genome) were resolved on a 1% agarose gel. The resolved nucleic acid was transferred to a positively charged nylon membrane Hybond-N+ (GE Healthcare, Chicago, IL, USA) and UV-crosslinked. The membrane was hybridized with a digoxygenin-labelled specific probe for the OpV1 full genome. The probe synthesis, hybridization and detection were obtained using the DIG High Prime DNA Labeling and Detection Starter Kit I (Roche, Indianapolis, IN, USA) according to the manufacturer’s instructions.

### 2.4. Phylogenetic and Pairwise Identity Analyses

Genome-wide pairwise nucleotide sequence identities between the 79 OpV1 genomes were determined using SDT v1.2 [61]. A genotype demarcation threshold of 95% was selected based on the distribution of pairwise identities and this revealed the existence of 15 genetically distinct OpV1 “genotype groups”.

Representative full-length nucleotide sequences from each of these 15 genotype groups, together with the genomes of geminiviruses belonging to the nine classified genera (30 sequences) and those that remained unassigned to a genus (12 sequences), were aligned by MAFFT v.7 [62]. This alignment was used to infer a Neighbor-Joining phylogenetic tree using a Jukes–Cantor substitution model with 1000 bootstrap replicates being used to test branch supports. Branches with < 60% bootstrap support were collapsed using TreeGraph2 [63], and the phylogenetic tree was midpoint-rooted.

The 79 OpV1 genomes were aligned with MAFFT v.7 [62] and the resulting alignment was used to infer a Neighbor-Joining phylogenetic tree using the Jukes–Cantor substitution model with 1000 bootstrap replicates being used to test branch supports. Branches with < 60% bootstrap support were collapsed using TreeGraph2 [63]. The OpV1 genome sequences, with recombination regions removed, were used to infer a Maximum-Likelihood (ML) phylogenetic tree using PHYML 3.0 [64] with the GTR+Γ+I substitution model selected as best fitting by jModelTest [65].

Datasets were also constructed that contained either the inferred Rep or inferred CP amino acid sequences of one, representative of each of the 15 OpV1genotype groups, along with representative sequences of viruses in the nine established geminivirus genera (30 viruses) and sequences from geminiviruses that remain unassigned to any genus (12 viruses). These Rep and CP amino acid datasets were aligned by MAFFT v.7 [62]. The alignments were used to infer ML phylogenetic trees using PHYML 3.0 [64] with the amino acid substitution models rtRev+G+F+I used for the CP dataset and rtRev+Γ+F+I used for the Rep dataset (these models were determined as best fitting by ProtTest; [66]), using the approximate likelihood ratio test (aLRT) of branch support. Branches with < 0.8 aLRT support were collapsed with TreeGraph2 [63] and both ML trees were rooted with sequences of viruses from the family *Genomoviridae*.

### 2.5. Capsid Protein Cluster Analysis

The CP amino acid sequences of all geminiviruses available in GenBank were extracted and clustered using CD-HIT [67] with a 90% identity threshold. A representative from each cluster was chosen and together with the CP amino acid sequences from representatives of the 15 OpV1 genotypes these were used to generate a sequence similarity network using the Enzyme Function Initiative–Enzyme Similarity Tool (EFI-EST) [68]. The network was created using a similarity score of 60 and E-value threshold of 1 × 10^−5^. The network was visualized in Cytoscape v3.7.1 [69] with the organic layout.

### 2.6. Recombination Analysis

The OpV1 genomes (*n* = 79) were aligned by MAFFT v.7 [62] and recombination analysis was performed by RDP4 v.4.97 [70] with default settings using the detection methods RDP [71], GENECONV [72], BOOTSCAN [73], MAXCHI [74], CHIMERA [75], SISCAN [76] and 3SEQ [77]. Recombination events that were detected by three or more methods with *p*-values < 0.05 were accepted as credible.

### 2.7. Virus Purification and Transmission Electron Microscopy

A total of 40 g of infected *N. benthamiana* leaves, 21 days post *Rhizobium*-mediated OpV1 infection, was homogenized in 40 mL of extraction buffer (1 × PBS pH 5.2, 10 mg/mL sodium ascorbate, 2 mM PMSF, 1 mM EDTA). The homogenate was filtered through two layers of cheese cloth and two layers of miracloth, and thereafter centrifuged for 30 min at 14,800× *g*. The clarified supernatant was kept at 4 °C overnight and then centrifuged twice for 30 min at 14,800× *g* and the pH was adjusted to 7.0. The supernatant was then centrifuged for 4 h at 32,000 rpm using a Beckman 32 Ti rotor, (Beckman Coulter, Pasadena, CA, USA) onto a 10% sucrose cushion and the pellet resuspended in 1 mL of 1× PBS. A total of 10 µL of a 1:10 dilution of the resuspended pellet was absorbed onto a carbon-coated copper grids for 10 min, washed, and negatively stained with 2% uranyl acetate. The grids were viewed using a Phillips CE 12 transmission electron microscope (Phillips, The Netherlands).

## 3. Results and Discussion

### 3.1. A Novel Cactus-Infecting Geminivirus

In an attempt to determine whether cacti are natural hosts of geminiviruses, we screened a total of 527 cactus samples from 18 countries for the presence of geminiviruses using an HTS approach. Most of the analyzed samples were collected in the USA (*n* = 394) from botanical gardens, herbaria and directly from native habitats. Based on geminivirus-like contigs recovered from these samples by HTS, a pair of abutting primers (OpV1 F/R) were designed to recover the full-length geminivirus-like genomes (or at least components of genomes). Amplicons of approximately 3 kb in length were produced using these primers from 31 cactus samples and nine cochineal insect samples.

Of the 31 samples found to contain geminivirus-like sequences, two cactus samples were from Mexico, 29 cactus samples were from the USA (Arizona, *n* = 28; Texas, *n* = 1), and all nine of the insect samples were from the USA. Of the areas in the USA where samples were collected, most (*n* = 20) were from the Desert Botanical Garden. Consequently, 25 additional non-cactus samples were collected from the Desert Botanical Garden to potentially identify alternate hosts. However, none of the non-cactus plant samples were found to contain OpV1-like sequences resembling those found in the cactus samples.

We amplified, cloned, and sequenced geminivirus genome-length DNA fragments (2940 to 2962 nt) from the 31 cactus, and nine insect samples that appeared to contain geminivirus-like DNA. These geminivirus-like genomes were tentatively named Opuntia virus 1 (OpV1), since most of them were retrieved from *Opuntia* spp. (Table 1). While some of the cochineal insects from which OpV1 genomes were recovered were collected from plants that also contained OpV1 genomes (*n* = 4) (Table 1), in other cases, insects containing OpV1 were collected from plants that did not detectably contain such genomes (*n* = 5) (Table 1).

Pairwise identity comparisons of OpV1 sequences to those of other known geminiviruses demonstrated that they all share < 64.9% genome identity with other known geminiviruses, and that all the OpV1 sequences share > 78.4% identity with one another (Supplementary Data 2 and 3).

**Figure 1:**
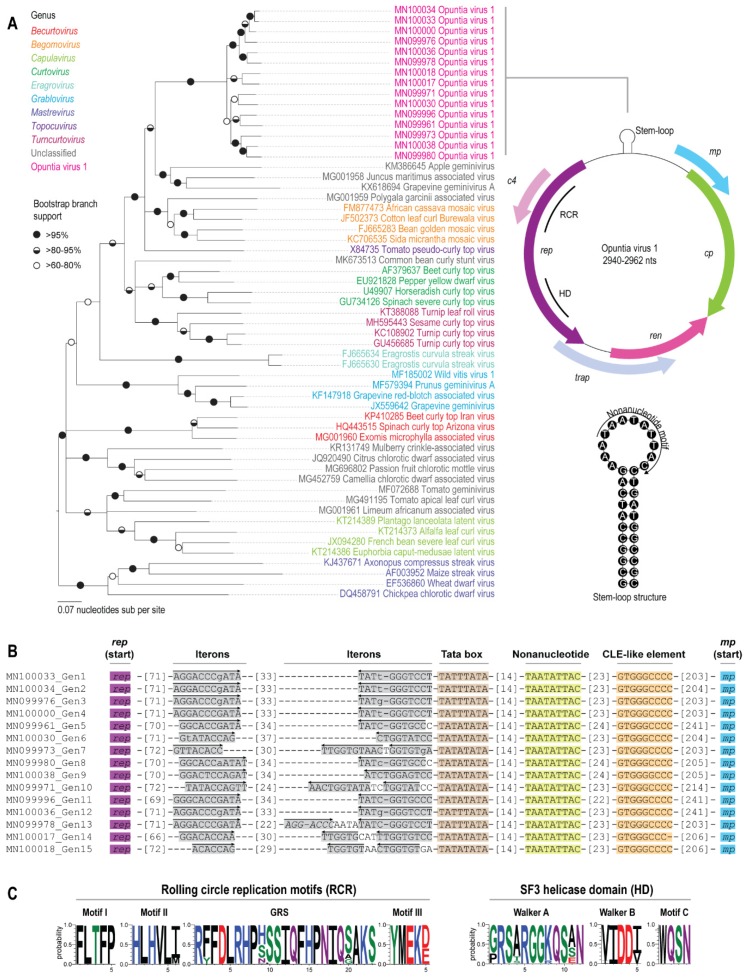
(**A**). Neighbor-Joining phylogenetic tree of the full-length genome of representatives of the 15 genotypes of OpV1 with those of the family *Geminiviridae*. Branches with < 60% bootstrap support are collapsed, and the tree is midpoint-rooted. The genomic organization of OpV1 and the stem–loop structure containing the nonanucleotide motif are shown to the right of the phylogenetic tree. (**B**). Nucleotide sequence and organization of origin of replication-associated iterative sequences “iterons” in the intergenic region. Arrows indicate the orientation of the iterons with respect to the nonanucleotide sequence. Lower-case letters in an iterated element indicate a nucleotide that does not match in all the iterons from OpV1 viruses of that same genotype. The TATA box, nonanucleotide motif and conserved late element (CLE)-like sequence. (**C**). Graphic representation of the variation in amino acids in the motifs from the SF3 helicase domains and the rolling circle replication motifs present in the Rep sequences of the 79 OpV1s.

OpV1 sequences all contain at least six recognizable open reading frames (ORFs) that were both capable of encoding proteins with >198 amino acids, and which shared some detectable similarity with known geminivirus-expressed proteins. If these ORFs are indeed genes, then the genome organization of the OpV1 sequences resembles that of viruses in the genus *Begomovirus* with monopartite genomes. On the presumed complementary strand, the OpV1 sequences potentially encode a replication-associated protein (Rep), a replication enhancer protein (Ren), a transactivation protein (TrAP) and a symptom determinant protein (C4) (Figure 1). A likely capsid protein (CP) and a possible movement protein (MP) are encoded on the virion strand. Within the OpV1 sequences, in the area corresponding to an intergenic region, there is a conserved nonanucleotide motif, “TAATATTAC”, contained within a likely stem–loop structure which, by analogy with other geminiviruses, is the likely site where virion strand replication is initiated (Figure 1). Within the intergenic region, we identified replication-associated iterative sequences “iterons”, the TATA box and conserved late element (CLE)-like sequences (Figure 1). There were two discernible iterons among most OpV1 isolates: a direct repeat adjacent to the *rep* gene TATA box, and an inverse repeat situated 41–42 nt upstream the TATA box. However, in a few OpV1 isolates, two in-tandem iterons are associated with the *rep* TATA box, similar to iterons observed in New World begomoviruses [78]. The specific sequence of the iterons also varied among OpV1 isolates, predominating those with a GGGTCC core sequence, although repeated elements with either GGTGCC, GGAGTC, GGTATY, or GGTGTC core sequences, among others, were also identified in some OpV1 isolates (Figure 1). The functional relevance of those differences is currently unknown. Another OpV1 feature is the position of the TATA box immediately adjacent to the *ori* stem-loop element (Figure 1), a unique arrangement among the geminiviruses.

As with the OpV1 nucleotide sequences, the amino acid sequences of the individual proteins that are likely encoded by these sequences display a considerable amount of diversity. Even the most conserved of these, CP and Rep, respectively, have pairwise amino acid sequence identities that are as low as 74.3% and 77.1% between different isolates.

Based on the distribution of pairwise nucleotide sequence identities shared by the 79 OpV1 sequences, a 95% sequence identity threshold was selected as a cut-off for defining distinct OpV1 genetic groupings. Applying this threshold to sub-classify the OpV1 sequences yielded 15 genotype groupings (Table 1).

It is noteworthy that, out of the 13 instances where more than one OpV1 sequence was isolated from a given plant sample, in seven cases the OpV1 sequences belonged to different genotype groups, i.e., in > 50% of instances where two different sequences were sampled from the same plant, these two sequences shared < 95% pairwise identity (Table 1). In three out of five instances where OpV1 sequences were retrieved from insects that were sampled on a plant from which OpV1 sequences were retrieved, the sequences in the insects were assigned to different genotypes than those to which the sequences in the plants were assigned.

Phylogenetic analysis of the full-length genome of OpV1 genotypes with representative geminivirus genome sequences (i.e., including representatives of the nine established geminivirus genera and other geminiviruses that have not yet been assigned to a genus) indicated that the OpV1 sequences could justifiably be assigned to a new geminivirus genus (Figure 1). The OpV1 sequences are most closely related to begomoviruses, topocuvirus and the unassigned geminiviruses Polygala garcinii associated virus (MG001959), apple geminivirus, (KM386645), Juncus maritimus associated virus (MG001958), and grapevine geminivirus A (KX618694).

**Figure 2:**
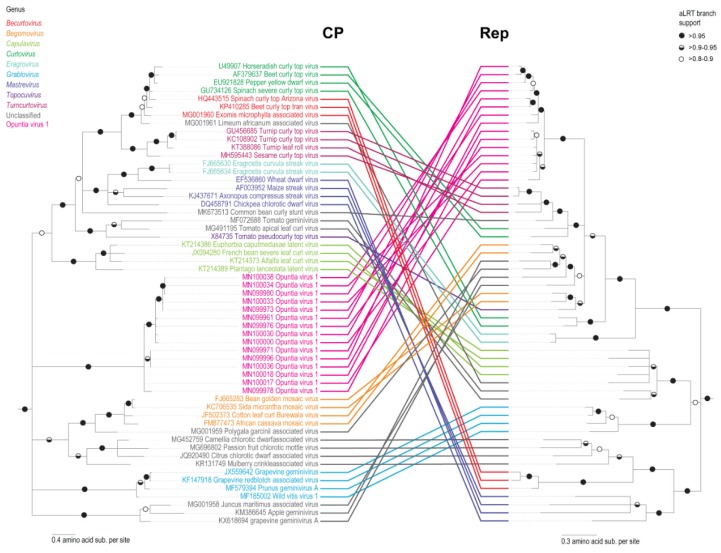
Maximum Likelihood (ML) phylogenetic trees of the Rep and CP amino acid sequences of the representative 15 genotypes of OpV1 together with other geminiviruses. Branches with aLRT support < 0.8 are collapsed and both trees were rooted with genomovirus [79] sequences.

Similarly, phylogenetic analysis of the predicted OpV1 Rep amino acid sequences, together with those of representative geminiviruses, indicated that the OpV1 Rep sequences are most closely related to those of begomoviruses, curtoviruses, topocuviruses, turncurtoviruses and the unclassified geminiviruses common bean curly stunt virus (MK673513)*;* Polygala garcinii associated virus (MG001959); apple geminivirus (KM386645); Juncus maritimus associated virus (MG001958) and grapevine geminivirus A (KX618694) (Figure 2). The OpV1 Rep amino acid sequences share < 68.2% identity with those of other geminiviruses.

The predicted OpV1 Rep amino acid sequences all contain predicted rolling circle replication, GRS, SF3 and Walker motifs that are similar to those found in other geminiviruses [80]. It is noteworthy that there is variability within these Rep motifs across the different predicted OpV1 Rep amino acid sequences, which further emphasizes the diversity within this group of viruses (Figure 1).

**Figure 3:**
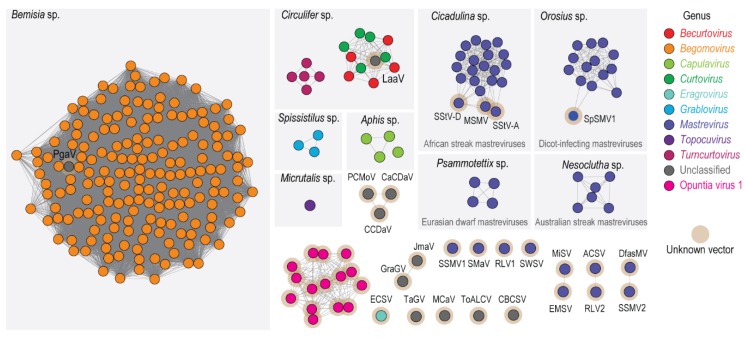
Sequence similarity network analysis of the CP amino acid sequences of representatives of the 15 genotypes from OpV1, together with those of the geminiviruses present in GenBank (dataset was created with an amino acid identity cut-off of 90%). The clusters are colored based on the genus or group. The genera that have known insect vectors are highlighted in a light grey box with the insect vector name displayed in the top. Clusters or singletons marked with a brown halo have no known insect vector associated with them. ACSV, Axonopus compressus streak virus; CaCDaV, Camellia chlorotic dwarf-associated virus; CCDaV, Camellia citrus chlorotic dwarf-associated virus; DfasMV, dragonfly-associated mastrevirus; CBCSV, common bean curly stunt virus; ECSV, Eragrostis curvula streak virus; EMSV, Eragrostis minor streak virus; GraGV, grapevine geminivirus; JmaV, Juncus maritimus-associated virus; LaaV, Limeum africanum-associated virus; MCaV, mulberry crinkle- associated virus; MiSV, Miscanthus streak virus; MSMV, maize streak Reunion virus; PCMoV, passion fruit chlorotic mottle virus; PgaV, Polygala garcinii-associated virus; RLV1, rice latent virus 1; RLV2, rice latent virus 2; SMaV, switchgrass mosaic-associated virus; SpSMV1, sweetpotato symptomless mastrevirus 1; SSMV1, Sporobolus striate mosaic virus 1; SSMV2, Sporobolus striate mosaic virus 2; SStV-A, sugarcane striate virus A; SStV-D, sugarcane striate virus D; SWSV, sugarcane white streak virus; TaGV, tomato-associated geminivirus; ToALCV, tomato apical leaf curl virus.

Unlike with the Rep amino acid sequences, the predicted OpV1 CP amino acid sequences group phylogenetically within a divergent clade (Figure 2). This is likely a consequence of the OpV1 CP amino acid sequences sharing < 28.9% amino acid identity with those of other geminiviruses. Recently, phylogenetic evidence that the CP amino acid sequences of geminiviruses are possibly co-diverging with their specific insect vectors has emerged [81]. A sequence similarity network analysis of the CP amino acid sequence of all geminiviruses (with a > 90% identity cut-off) was generated and the association of the known geminivirus CPs with known insect vectors is summarized in Figure 3. It is clear that, whenever geminiviruses share an insect vector, their CP amino acid sequences cluster together. As expected, given the divergence of OpV1 CP amino acid sequences relative to those of other geminiviruses, these sequences form their own cluster, implying that they are likely to be transmitted by an insect species that has not previously been associated with geminivirus transmission. Given the association of cochineal insects with the cactus plants from which OpV1 sequences were isolated and the direct isolation of OpV1 sequences from some of these insects, it remains plausible that these insects may be OpV1 transmission vectors. However, controlled insect transmission experiments will be needed to properly test this hypothesis.

The high degree of nucleotide sequence diversity amongst the OpV1 sequences suggests, assuming a similar rate of nucleotide sequence diversification to that seen in other geminiviruses, that OpV1 has likely been circulating in the USA for more than 600 years, i.e., the approximate time it would take mastrevirus and begomovirus species to achieve the degree of diversity observed for the OpV1 sequences [82,83,84,85,86,87]. The lower numbers of OpV1-positive samples found outside the USA certainly represents a sampling bias. Although the number of OpV1-positive cactus plants originating from Mexico were very low (2/31 tested plants), this 6.4% prevalence is not substantially different to the 7.4% OpV1 prevalence in cactus samples from the USA.

### 3.2. Testing the Infectivity of the Novel Cactus-Infecting Geminiviruses

To assess the infectivity of OpV1, a *Rhizobium*-infectious clone was created using the isolate DBG_14_1 (MN100000). The infectious clone was generated using ~1.9 unit length DBG_14_1 sequences cloned tandem within the pGTV-kan binary vector [60]. *Rhizobium*-mediated inoculation assays were performed on plants of *N. benthamiana*, *O. ficus-indica*, *O. microdasys*, *O. engelmannii* and *O. santa-rita*. The experiments with *N. benthamiana* were carried out in triplicate, with two replicates consisting of 18 inoculated plants and the third of seven inoculated plants; two negative control (non-inoculated) plants were included in all experiments. The rate of infection in *N. benthamiana* plants varied between replicates. In the initial experiment, five out 18 plants were positive for OpV1 infection and systemic viral infection could be detected at 5 days post inoculation (dpi). In the second experiment, 10 out of 18 plants were positive for OpV1, with systemic viral infection also being detectable at 5 dpi. In the third experiment, samples were only evaluated at 21 dpi and they were all positive for OpV1 by PCR. The Southern blot analysis of the third infectivity assay of *Rhizobium*-mediated OpV1-infected *N. benthamiana* plants corroborated with the PCR results, showing the viral DNA replicative forms in all seven inoculated plants (open circular, linear, covalent closed circular and single stranded) and no viral infection in the negative controls (Supplementary Figure S1). We also observed geminate particles in the viral extract of the *Rhizobium*-mediated OpV1 infected *N. benthamiana* leaves (Supplementary Figure S1). In the inoculation assays with *O. ficus-indica*, *O. microdasys*, *O. engelmannii* and *O. santa-rita,* five plants (one individual pad) from each species were *Rhizobium*-infiltrated and one plant was kept as a negative control (non-inoculated). Cacti are perennial plants that have slow growth rates, and it is therefore difficult to assess systemic infection. From the inoculated cactus species, only *O. ficus-indica* and *O. microdasys* plants developed new pads over the course of the experiment (~8 months). Wherever new pads were unavailable for sampling, the area of sampling in the originally inoculated pad was selected to be as distant as possible from the spot where the *Rhizobium*-inoculation was carried out. Of the 16 *Opuntia* plants inoculated with OpV1, only one, an individual of *O. microdasys*, was positive for OpV1 by PCR five months post-inoculation.

No symptoms associated with viral infection were observed in either *N. benthamiana* or *O. microdasys.* Infections were further confirmed by recovering viral genomes (which were cloned and sequenced; Supplementary Data 4) from the non-inoculated leaves of five *N. benthamiana* OpV1 positive plants and one OpV1 positive *O. microdasys* plant.

### 3.3. Evolutionary Dynamics of the Novel Divergent Geminivirus Group

Given that genetic recombination has been found to occur frequently during the evolution of other geminiviruses and that recombination has been implicated in the genesis of at least four of the currently recognized geminivirus genera [43,46,88,89,90,91,92,93,94,95,96], we examined the OpV1 sequences for evidence of recombination. In total, we detected 23 well-supported recombination events during the evolution of the 79 OpV1 sequences from their most recent common ancestor. The sizes of genome fragments transferred during these recombination events ranged from approximately 64 to 1171 nt (Table 2). Except for the only sequence belonging to genotype 8, all the sequences displayed well supported evidence of at least one recombination event. Some of the OpV1 sequences assigned to genotype 12 display evidence of at least five distinct recombination events. While some of the detected recombination events appear to have occurred quite recently, in that they were only detectable within single OpV1 sequences, others, such as one event that is detectable in all of the genotype 1, 2, 12 and 13 sequences, likely occurred in the more distant past, i.e., prior to the time when the most recent common ancestors of the sequences, sharing evidence of the recombination events, existed.

The largest genome fragment transferred during the detected recombination events was seen in the genotype 6 sequences, and involved the transfer of the ~40% of the genome spanning the intergenic region and the virion strand protein-coding genes.

**Figure 4:**
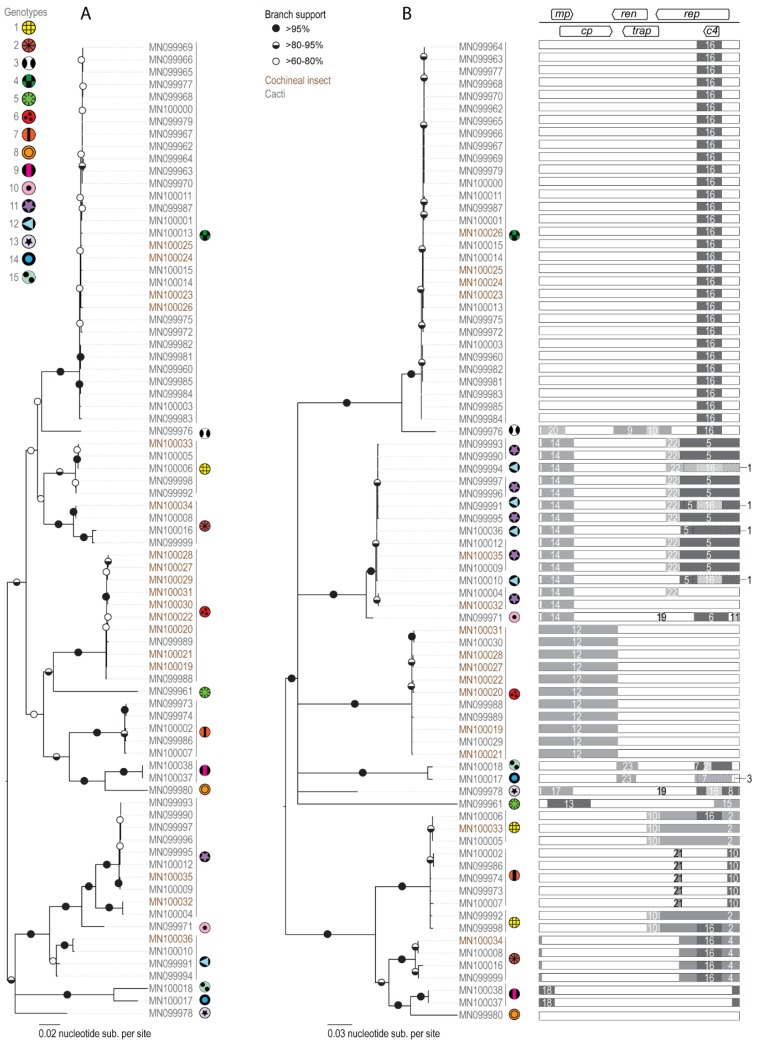
(**A**). Neighbor-Joining phylogenetic tree of the 79 OpV1 genomes recovered in this study. Branches with <60% bootstrap support are collapsed. (**B**). Maximum Likelihood phylogenetic tree of the 79 OpV1 genomes with recombination regions removed. For each genome, a graphic on the right indicates the recombination event with its breakpoint location within the genome. Branches with <60% bootstrap support are collapsed. The 15 genotypes are marked with symbols and genomes that have been recovered from plants (accession numbers in grey) and cochineal insects (accession numbers in brown) are indicated.

As has been previously noted for other geminiviruses [95,96,97,98], a high proportion of the detected recombination events have breakpoints in the intergenic region at or close to the presumed virion strand origin of replication. Similar to breakpoint patterns seen in other geminiviruses, the Rep/AC4 region of the genome appears to be the genome site outside the intergenic region where recombination breakpoints most frequently occur (Figure 4). Conversely, the region of the genome spanning the *ren* and *trap* genes appears to have the lowest frequency of detectable recombination breakpoints.

### 3.4. Identification of Sub/Super- Genomic Molecules

**Figure 5:**
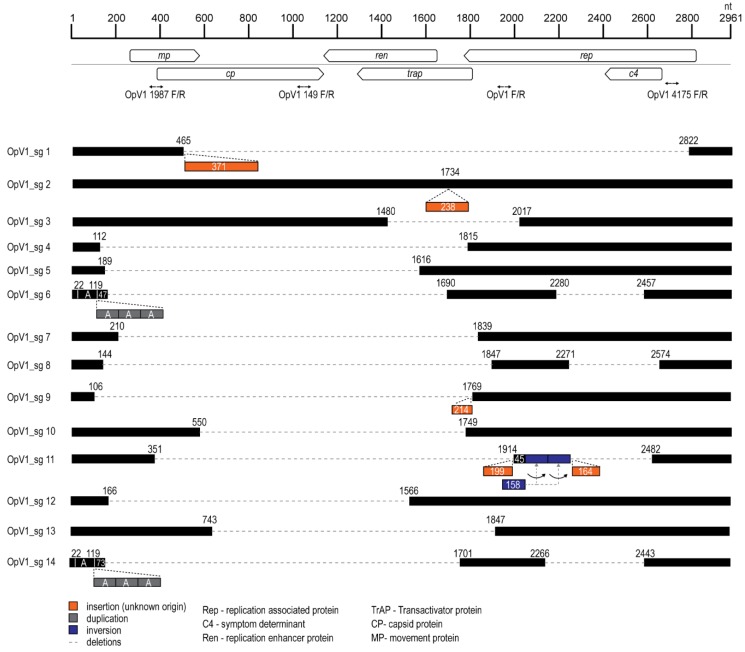
Structure of OpV1 sub/super-genomic molecules in comparison to an OpV1 parental full-length genome. The areas where deletion occurred are presented by dotted grey lines, insertions, inversions and duplications are represented by orange, blue and grey boxes respectively. The primers pairs used to obtain the sub/super-genomic molecules are shown at their respective binding sites on the parental full-length genome.

It is noteworthy that during attempts to clone OpV1 sequences, we recovered 12 apparently sub-genome length clones containing OpV1 sequences from nine cactus plants and one cochineal insect (OpV1 sg 9) (Figure 5), as well as a sequence containing a full complement of OpV1 DNA together with a 238 nt long sequence insert of unknown origin (i.e., super-genome length) from one cactus plant (OpV1 sg 2). The presence of similar sub-genome length geminivirus-derived DNA within geminivirus infections, commonly referred to as sub-genomic molecules, have been extensively reported elsewhere [27,99,100,101,102,103,104,105,106]. In addition to deletions, in some cases sub-genomic molecules have also been found to contain sequence insertions, duplications and inversions [103,107,108]. The conservation within sub-genomic molecules of intergenic region sequences—the portion of geminivirus genomes containing the origin of virion-strand replication—indicates that these molecules are, in many cases, potentially either self-replication-competent (if they contain an intact *rep* gene) or are capable of being trans-replicated by non-defective viruses [109].

From the three cactus samples that we examined (one each of *O. spinosibacca*, *O. rufida* and *O. santa-rita*), we were only able to recover sub-genomic molecules (Table 1). None of these sub-genomic molecules (OpV1 sg 10, -11, -13 and -14; Figure 5) had a *rep* gene without disruption, which indicates that they would have needed to be trans-replicated by either a non-defective OpV1 variant or some other geminivirus.

OpV1 sg 6 and -14, which were each recovered from different cactus plants, displayed an interesting similarity. Both contain three tandem repeats of the portion of the intergenic region between 22 and 119 nt upstream of the presumed virion strand origin of replication (Figure 5). OpV1 sg 6, -8, -11 and -14 all have a similar domain deleted within the Rep coding region (Figure 5). The deletions in the Rep-coding region in these molecules are such that the N-terminus of the Rep amino acid sequence has at least two intact rolling circle replication motifs (motif I and II). OpV1 sg 6, -8 and -14 have a second ORF that has an in-frame C-terminus with two helicase motifs (Walker B and motif C). Furthermore, all OpV1 sg molecules except sg 2 and -3 have a deletion spanning the region (743–1566 nt) that encodes the CP, Ren and TrAP proteins (Figure 5).

Only three of the 14 sub-genomic molecules (OpV1 sg 2, -5, and -12) have an intact Rep coding region and only two have an intact CP coding region (OpV1 sg 2 and -3).

OpV1 sg 2, is larger than the predicted full-length genome of OpV1 with an insert of 238 nt of unknown origin (we have labelled this as super-genomic molecule), and has all coding regions intact, except that encoding the Ren protein (Figure 5). Insertions of unknown origin are in OpV1 sg 1 (371 nt), OpV1 sg 9 (214) and OpV1 sg 11 (199/164 nt). In a recent study on beets (*Beta vulgaris*) infected with the geminivirus beet curly top Iran virus (BCTIV), circular molecules labelled as “minicircles” were identified containing large AT-rich host derived sequences, as well as the BCTIV intergenic region containing the origin of replication [110]. The minicircles have been proposed to act as a possible mechanism of horizontal gene transfer among host plants. It is important to highlight that the actual diversity of sub/super-genomic DNA molecules that might arise during OpV1 infections is likely higher than those that we have detected here, since the PCR primer binding sites used to amplify these molecules may impact the distributions of the observed deleted regions.

The mechanisms that generate geminivirus sub-genomic molecules are still unclear, although the presence of secondary structures and possible clashes between the replication and transcriptional machinery (due to the bidirectionality of transcription and replication in these viruses) have been suggested as facilitators in this process [111]. Some geminivirus sub-genomic molecules have been shown to be packaged into virions and transmitted by their insect vectors [100,112]; in some cases they can be co-transmitted with their helper/original virus [103,109] which supports our findings of sub-genomic molecules (OpV1 sg 9) along with full-length OpV1 genomes in the cochineal insect (Table 1). The sequence of the sub/super-genomic molecules are provided in Supplementary Data 5.

## 4. Concluding Remarks

OpV1, the first reported cactus-infecting DNA virus, is the latest member of the family *Geminiviridae* that will likely require assignment to a novel genus. Despite its high degree of divergence relative to other known geminiviruses—particularly in the CP—OpV1 has numerous similarities with its nearest geminiviruses relatives. OpV1 has a genome organization that is very similar to that of other geminiviruses; it displays patterns of recombination that mirror those of other geminiviruses, and it forms sub-genomes with patterns of deletion and sequence insertions and rearrangements that are reminiscent of those formed by other geminiviruses.

OpV1 appears to be restricted to the family Cactaceae but has a broad range of host species within the family. In the cactus samples collected to date, no specific host species association to any OpV1 genotype groupings could be inferred. In some cases, we recovered up to five different genotypes from one cactus species and genotype 6 was recovered from 11 different cacti. OpV1 genomes were recovered only from cactus plants in the USA (proportion of plants tested that were positive 7.4%) and Mexico (proportion of plants tested that were positive 6.5%). We were unable to conclusively determine whether cochineal insects are a transmission vector of OpV1, however, it is evident that these insects do acquire the virus upon feeding on infected cactus plants, and therefore it is plausible that they could transmit the virus. Additionally, the host range of cochineal insects is restricted to cacti, further supporting their role as vectors of OpV1, which, to date, has only been identified as infecting cacti. This is also well supported by the CP cluster analyses where the OpV1 CPs form a distinct cluster from those geminiviruses with known insect vectors. Further, we were able to show that cloned OpV1 sequences are capable of initiating asymptomatic systemic infections in *N. benthamiana* and *O. microdasys*. Although we can only confirm that OpV1 is present in the USA and Mexico, it remains plausible that it occurs in other areas of the Americas or parts of the world where cacti are cultivated for agricultural or ornamental purposes.

## Figures and Tables

**Table 1:** Summary of the isolates, accession number, genotype, host species, collection dates and country from which the 79 genomes of the Opuntia virus 1 has been recovered. Details of the cochineal insects sampled and identification of sub/super-genomic molecules from specific plants are listed.

Virus	Isolate	Accession Number	Genotype	Host species	Sampling Year	Region of Collection	Associated Insect Samples	Sub/Super-Genomic
OpV1	2013_1	MN099960	4	*Lophocereus schottii*	2015	Arizona, USA		
OpV1	2013_2	MN099981	4		2015	Arizona, USA		
OpV1	2013_3	MN099982	4		2015	Arizona, USA		
OpV1	2014_1	MN099983	4	*Opuntia stenopetala*	2015	Arizona, USA		
OpV1	2014_2	MN099984	4		2015	Arizona, USA		
OpV1	2014_3	MN099985	4		2015	Arizona, USA		
OpV1	2014_4	MN099986	4		2015	Arizona, USA		
OpV1	2014_5	MN099987	4		2015	Arizona, USA		
OpV1	ASU_PP2	MN099961	5	*Cylindropuntia fulgida*	2018	Arizona, USA		
OpV1	ASUH_12	MN099962	4	*Opuntia tapona*	2002	Baja California, Mexico		
OpV1	ASUH_16	MN099963	4	*Opuntia engelmannii*	2010	Arizona, USA		
OpV1	ASUH_20	MN099964	4	*Opuntia santa-rita*	2002	Sonora, Mexico		
OpV1	Cacti_2_1	MN099988	6	*Opuntia santa-rita*	2017	Arizona, USA	SI_7	
OpV1	Cacti_2_2	MN099989	6		2017	Arizona, USA		
OpV1	DBG10_5	MN099990	11	*Opuntia cespitosa*	2017	Arizona, USA		OpV1 sg-1
OpV1	DBG10_9	MN099991	12		2017	Arizona, USA		OpV1 sg-3
OpV1	DBG10_149	MN099992	11		2017	Arizona, USA		
OpV1	DBG10_1972	MN099993	11		2017	Arizona, USA		
OpV1	DBG10_2558	MN099994	12		2017	Arizona, USA		
OpV1	DBG10_2562	MN099995	11		2017	Arizona, USA		
OpV1	DBG13_5	MN099996	11	*Opuntia basilaris*	2017	Arizona, USA		OpV1 sg-2
OpV1	DBG13_9	MN099997	11		2017	Arizona, USA		OpV1 sg-4
OpV1	DBG13_1987	MN099998	1		2017	Arizona, USA		
OpV1	DBG_14_1	MN100000	4	*Opuntia echios* var. echios	2017	Arizona, USA	SI_1	
OpV1	DBG_14_2	MN100001	4		2017	Arizona, USA		
OpV1	DBG_14_3	MN100002	7		2017	Arizona, USA		
OpV1	DBG_14_4	MN100003	4		2017	Arizona, USA		
OpV1	DBG_46	MN100013	4		2018	Arizona, USA		
OpV1	DBG_47	MN100014	4		2018	Arizona, USA		
OpV1	DBG_48	MN100015	4		2018	Arizona, USA		
OpV1	DBG_26	MN100004	11	*Opuntia rufida*	2018	Arizona, USA		OpV1 sg-6
OpV1	DBG_31_1	MN100005	1	*Opuntia mackensenii*	2018	Arizona, USA		
OpV1	DBG_31_2	MN100006	1		2018	Arizona, USA		
OpV1	DBG34	MN099999	2	*Opuntia robusta*	2018	Arizona, USA	SI_33	OpV1 sg-8
OpV1	DBG_34	MN100007	7		2018	Arizona, USA		
OpV1	DBG_36	MN100008	2	*Opuntia englemannii x.O. rufida*	2018	Arizona, USA	SI_35	
OpV1	DBG_38	MN100009	11	*Opuntia martiniana*	2018	Arizona, USA		
OpV1	DBG_41	MN100010	12	*Opuntia rooneyi*	2018	Arizona, USA		OpV1 sg-5
OpV1	DBG_42_1	MN100011	4	*Opuntia englemannii*	2018	Arizona, USA		OpV1 sg-12
OpV1	DBG_42_2	MN100012	11		2018	Arizona, USA		
OpV1	DBG_42_3	MN099971	10		2018	Arizona, USA		
OpV1	DBG_56	MN099972	4	*Opuntia basilaris*	2018	Arizona, USA		
OpV1	DBG_57	MN099973	7		2018	Arizona, USA		
OpV1	DBG_57_2	MN099974	7		2018	Arizona, USA		
OpV1	DBG_58	MN099975	4		2018	Arizona, USA		
OpV1	DBG_72	MN099976	3	*Opuntia rufida*	2018	Arizona, USA		
OpV1	DBG74	MN099965	4	*Opuntia robusta*	2018	Arizona, USA		
OpV1	DBG75	MN099966	4	*Opuntia basilaris*	2018	Arizona, USA		
OpV1	DBG80	MN099967	4	*Cylindropuntia echinocarpa*	2018	Arizona, USA		
OpV1	DBG86	MN099968	4	*Cylindropuntia spinosior*	2018	Arizona, USA		
OpV1	DBG_86	MN099977	4		2018	Arizona, USA		
OpV1	DBG88	MN099969	4	*Opuntia cf polyacantha*	2018	Arizona, USA		
OpV1	DBG90	MN099970	4	*Opuntia phaeacantha*	2019	Arizona, USA		
OpV1	LCM_85	MN100016	2	*Opuntia aureispina*	2015	Texas, USA		
OpV1	LCM_91_1	MN100017	14	*Cylindropuntia arbuscula*	2015	Arizona, USA		OpV1 sg-7
OpV1	LCM_91_2	MN100018	15		2015	Arizona, USA		
OpV1	S18_1	MN099978	13	*Opuntia engelmannii*	2018	Arizona, USA		
OpV1	S18_8	MN099979	4	*Opuntia santa-rita*	2018	Arizona, USA		
OpV1	S18_89	MN099980	8	*Opuntia engelmannii*	2018	Arizona, USA		
OpV1	TM_cacti_2_1	MN100037	9	*Opuntia engelmannii.*	2018	Arizona, USA		
OpV1	TM_cacti_2_2	MN100038	9		2018	Arizona, USA		
OpV1	SI_0_1	MN100019	6	*Dactylopius* sp.	2017	Arizona, USA		
OpV1	SI_0_2	MN100020	6		2017	Arizona, USA		
OpV1	SI_0_3	MN100021	6		2017	Arizona, USA		
OpV1	SI_0_4	MN100022	6		2017	Arizona, USA		
OpV1	SI_1_1	MN100023	4	*Dactylopius* sp.	2017	Arizona, USA	DBG14	
OpV1	SI_1_2	MN100024	4		2017	Arizona, USA		
OpV1	SI_1_3	MN100025	4		2017	Arizona, USA		
OpV1	SI_1_4	MN100026	4		2017	Arizona, USA		
OpV1	SI_7_1	MN100027	6	*Dactylopius* sp.	2017	Arizona, USA	Cacti 2	
OpV1	SI_7_2	MN100028	6		2017	Arizona, USA		
OpV1	SI_7_3	MN100029	6		2017	Arizona, USA		
OpV1	SI_9_1	MN100030	6	*Dactylopius* sp.	2017	Arizona, USA		
OpV1	SI_9_2	MN100031	6		2017	Arizona, USA		
OpV1	SI_23	MN100032	11	*Dactylopius* sp.	2018	Arizona, USA		
OpV1	SI_28	MN100033	1	*Dactylopius* sp.	2018	Arizona, USA		
OpV1	SI_33	MN100034	2	*Dactylopius* sp.	2018	Arizona, USA	DBG34	OpV1 sg-9
OpV1	SI_35	MN100035	11	*Dactylopius* sp.	2018	Arizona, USA	DBG36	
OpV1	SI_39	MN100036	12	*Dactylopius s*p.	2018	Arizona, USA		
	DBG_28			*Opuntia spinosibacca*	2018	Arizona, USA		OpV1 sg-14
	DBG_69			*Opuntia rufida*	2018	Arizona, USA		OpV1 sg-13
	S18_9			*Opuntia santa-rita*	2018	Arizona, USA		OpV1 sg-10
								OpV1 sg-11

**Table 2:** Summary of the 23 events of recombination detected by RDP4. The methods used to detect recombination are RDP (R) GENCONV (G), BOOTSCAN (B), MAXCHI (M), CHIMERA (C), SISCAN (S) and 3SEQ (T). The method with the highest *p*-value for each recombination event is bolded. Sites where the actual breakpoint is undetermined are marked with *. (tr) denotes trace of recombination signal.

Recombination Event	Region	Recombinant Sequence(s)	Minor Parental Sequence(s)	Major Parental Sequence(s)	Detection Methods	*p*-Value
1	2156–4	Genotype 12	Genotype 2	Genotype 11	RGBMCST	1.41 × 10^−69^
2	1915–2	Genotype 1	Genotype 4	Genotype 7	RGBMCST	4.11 × 10^−56^
3	2338–2878	Genotype 14	Genotype 2	Genotype 15	RBMCS	1.74 × 10^−34^
			Genotype 7			
4	2066–42	Genotype 2	Genotype 4	Genotype 9	RGBMCS	9.95 × 10^−39^
				Genotype 7		
5	2088–2961	Genotype 11	Genotype 8	Genotype 10	RGBMCS	6.08 × 10^−29^
		Genotype 12 (tr)	Genotype 5			
6	2304–2819	Genotype 10	Genotype 6	Genotype 11	RGBMCST	1.08 × 10^−24^
7	2301–2844	Genotype 15	Genotype 7	Genotype 11	RGBMCS	4.94 × 10^−17^
		Genotype 14 (tr)		Genotype 4		
8	2333–2939	Genotype 13	Genotype 4	Genotype 11	RGBMCS	7.33 × 10^−29^
				Genotype 7		
9	1088–1957	Genotype 3	Genotype 7	Genotype 4	RGBMCST	1.17 × 10^−24^
10	1582–1765	Genotype 3	Genotype 13	Genotype 2	RBCS	2.05 × 10^−13^
		Genotype 1		Genotype 4		
		Genotype 7				
11	2860–2946	Genotype 10	Genotype 6	Genotype 11	RGMCST	2.04 × 10^−10^
12	2928–1156	Genotype 6	Genotype 4	Genotype 9	RBMCS	6.50 × 10^−26^
			Genotype 3	Genotype 11		
				Genotype 13		
				Genotype 12		
13	514–1166	Genotype 5	Genotype 4	Genotype 11	RGBMCS	6.12 × 10^−18^
			Genotype 3			
14	31 *–512	Genotype 10	Genotype 3	Genotype 14	RBMC	4.05 × 10^−9^
		Genotype 12 (tr)	Genotype 7	Genotype 9		
		Genotype 11 (tr)				
15	1494 *–1866	Genotype 5	Genotype 6	Genotype 11	RGBMCST	2.93 × 10^−10^
				Genotype 10		
				Genotype 12		
16	2332–2719	Genotype 4	Unknown	Genotype 6	RGBMCST	1.88 × 10^−32^
		Genotype 13				
		Genotype 2				
		Genotype 12				
		Genotye1				
17	30 *–489	Genotype 13	Genotype 3	Genotype 14	GBMCST	9.73 × 10^−9^
				Genotype 15		
18	2846 *–216	Genotype 9	Genotype 15	Genotype 7	RMC	1.71 × 10^−8^
			Genotype 14			
19	1791–1855	Genotype 10	Genotype 2	Genotype 12	GBT	9.66 × 10^−6^
		Genotype 13 (tr)		Genotype 11		
20	27–373	Genotype 3	Genotype 7	Genotype 4	RBMCT	5.80 × 10^−6^
			Genotype 1			
21	2445 *–2543	Genotype 15	Genotype 11	Unknown	RGBC	1.77 × 10^−05^
		Genotype 7	Genotype 8			
22	1881–26 *	Genotype 12	Genotype 4	Genotype 14	GBMCST	8.71 × 10^−5^
		Genotype 11 (tr)		Genotype 15		
23	1132–1462	Genotype 15	Unknown	Genotype 12	MCT	5.90 × 10^−3^
		Genotype 14		Genotype 11		

## Supplementary Materials

Supplementary materials can be found at https://www.mdpi.com/1999-4915/12/4/398/s1.
