# Supplementary figures and images for: A Novel Divergent Geminivirus Identified in Asymptomatic New World Cactaceae Plants
Source: Viruses. 2020 Apr 3;12(4):398. doi: 10.3390/v12040398 (PMC7232249; doi:10.3390/v12040398)

**A**

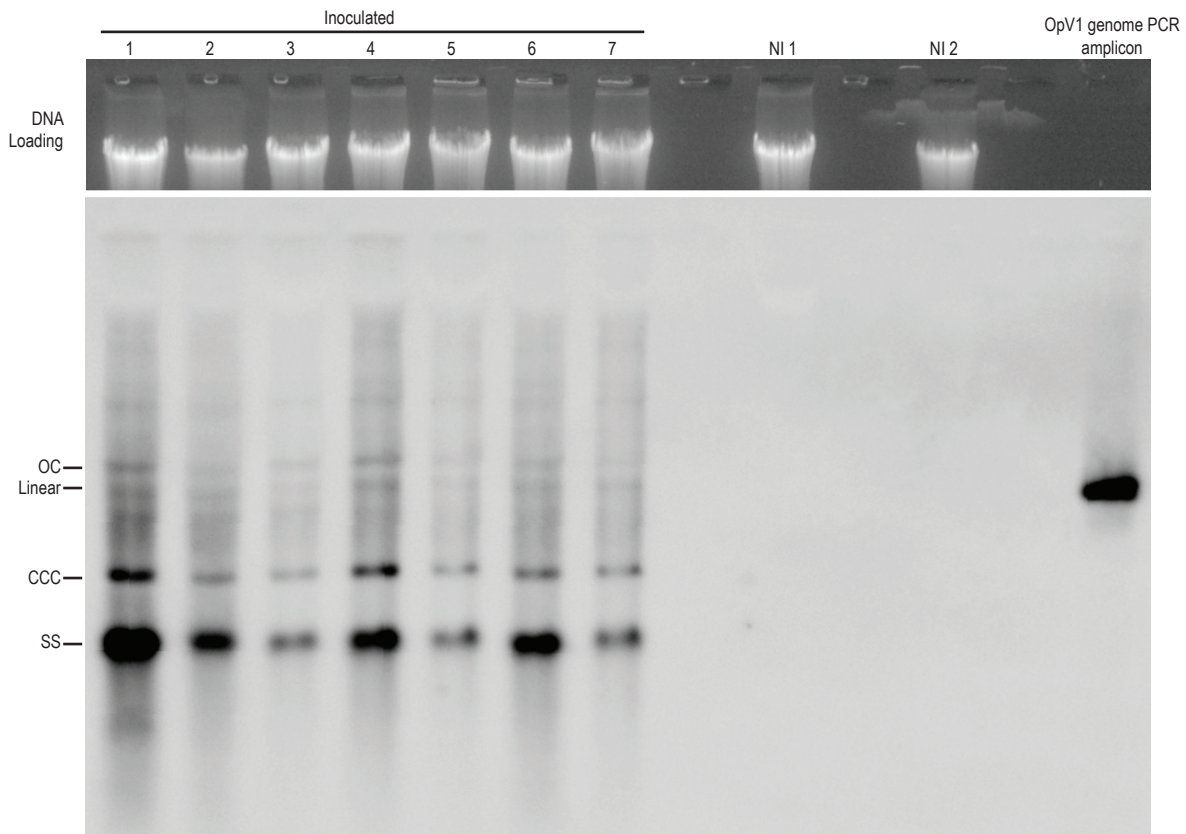**B**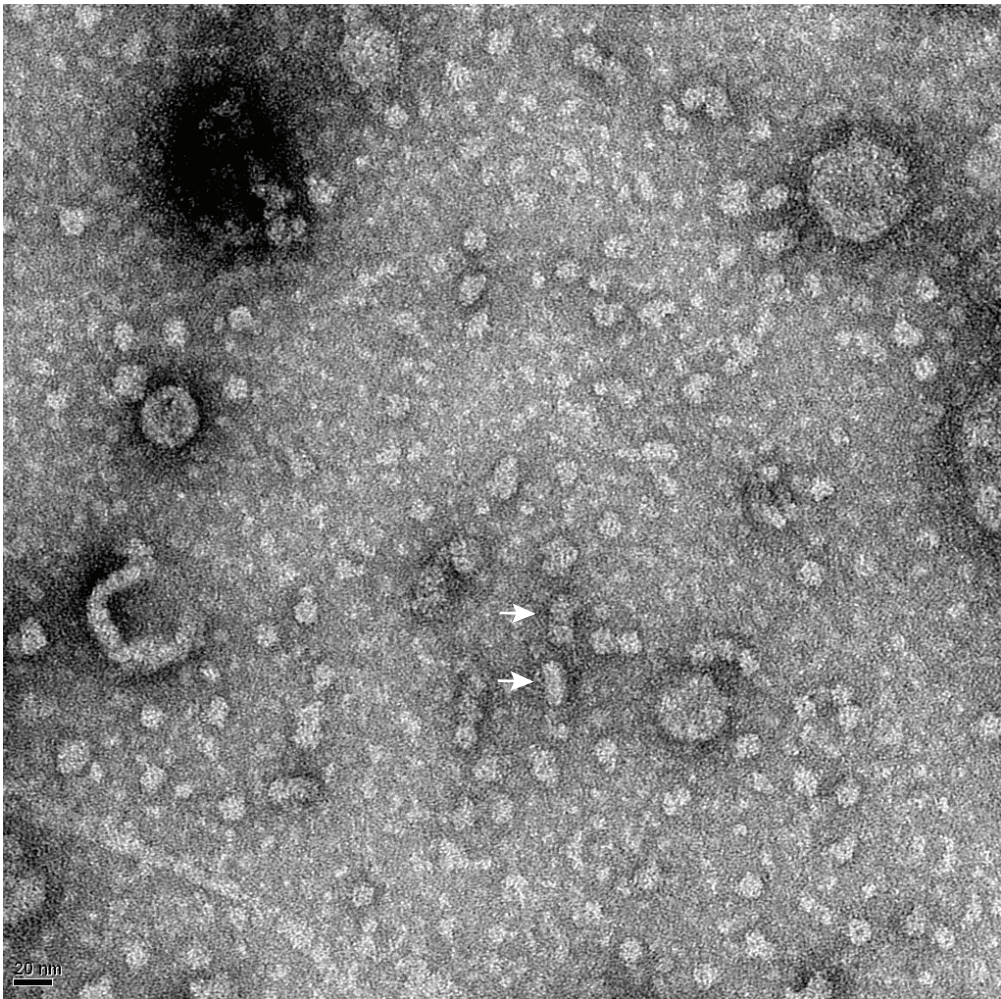

Supplement: Supplementary file 1 [file viruses-12-00398-s001.zip › SupplementaryFigure1.pdf]
